# Convergence theorems for generalized nonexpansive multivalued mappings in hyperbolic spaces

**DOI:** 10.1186/s40064-016-2557-y

**Published:** 2016-06-29

**Authors:** Jong Kyu Kim, Ramesh Prasad Pathak, Samir Dashputre, Shailesh Dhar Diwan, Rajlaxmi Gupta

**Affiliations:** Department of Mathematics Education, Kyungnam University, Changwon, Gyeongnam 51767 Korea; Department of Applied Mathematics, National Institute of Technology, Raipur, 492001 India; Department of Applied Mathematics, Shri Shankaracharya Group of Institutions, Junwani, Bhilai 490020 India; Department of Mathematics, Sant Guru Ghasidas, Govt. P. G. College, Kurud, Dhamtari 493663 India

**Keywords:** Generalized nonexpansive multivalued mappings, Iteration process, Strong and $\Delta$-convergence theorems, Hyperbolic spaces, 47H09, 47H10

## Abstract

In this paper, we establish the existence of a fixed point for generalized nonexpansive multivalued mappings in hyperbolic spaces and we prove some $\Delta$-convergence and strong convergence theorems for the iterative scheme proposed by Chang et al. (Appl Math Comp 249:535–540, 2014) to approximate a fixed point for generalized nonexpansive multivalued mapping under suitable conditions. Our results are the extension and improvements of the recent well-known results announced in the current literature.

## Background

The study of fixed points for multivalued contractions and nonexpansive mappings using the Hausdorff metric was initiated by Markin (1973) and Nadler (1969). The existence of fixed points for multivalued nonexpansive mappings in convex metric spaces has been shown by Shimizu and Takahashi (1996), that is, they proved that every multivalued mapping $T : X \rightarrow C(X)$ has a fixed point in a bounded, complete and uniformly convex metric space (*X*, *d*), where *C*(*X*) is the family of all compact subsets of X.

Late, so many fixed point theorems for such mappings have applications in control theory, convex optimization, differential inclusion and economics (see Gorniewicz 1999 and references cited therein).

Since then many authors have been published papers on the existence and convergence of fixed points for multivalued nonexpansive mappings in uniformly convex Banach spaces and convex metric spaces.

The theory of multivalued nonexpansive mappings is more difficult than the corresponding theory of single valued nonexpansive mappings. Different iterative algorithms have been used to approximate the fixed points of multivalued nonexpansive mappings (see Abbas et al. 2011; Khan and Yildirim 2012; Khan et al. 2010; Panyanak 2007; Shahzad and Zegeye 2009; Sastry and Babu 2005; Song and Wang 2008, 2009; Song and Cho 2011) in uniformly convex Banach spaces.

On the other hand, in García-Falset et al. (2011) introduced two new conditions on single valued mappings, are called condition (*E*) and $(C_{\lambda })$ which are weaker than nonexpansive and stronger than quasi-nonexpansive.

Recently, Abkar and Eslamain (2012) introduced an iterative process for a finite family of generalized nonexpansive multivalued mappings and proved $\Delta$-convergence and strong convergence theorems in CAT(0) spaces.

In the view of the fact, many researchers motivated towards to introduced various numbers of iterative processes for approximating fixed points and established $\Delta -$convergence and strong convergence theorems, not only for multivalued nonexpansve mappings but also its general class of multivalued mappings in CAT(0) spaces (see Abkar and Eslamain 2012; Dhompongsa et al. 2012; Eslamian and Abkar 2011; Khan et al. 2011; Laowang and Panyanak 2009; Nanjaras et al. 2010; Pathak et al. 2015) and in hyperbolic spaces (see Chang et al. 2014a, b, 2015; Khan and Abbas 2014).

The purpose of this paper is to establish existence of a fixed point for generalized nonexpansive multivalued mappings in the setting of hyperbolic spaces. Under suitable conditions some $\Delta$-convergence and strong convergence theorems for the iterative scheme proposed by Chang et al. (2014a) to approximate a fixed point for generalized nonexpansive multivalued mapping are proved. Our results are the extensions and improvements of the recent well-known results announced in the current literature.

## Preliminaries

Throughout in this paper we consider hyperbolic space which is introduced by Kohlenbach (2005) as follows:

A hyperbolic space (*X*, *d*, *W*) is a metric space (*X*, *d*) together with a convexity mapping $W: X^{2} \times [0,1] \rightarrow X$ satisfying

- $$(W_{1})\quad d( u, W(x, y, \alpha )) \le \alpha d( u, x) + ( 1-\alpha ) d( u, y);$$
- $$(W_{2}) \quad d(W(x, y, \alpha ), W(x, y,\beta )) = \vert \alpha - \beta \vert d( x, y); $$
- $$(W_{3}) \quad W(x, y, \alpha ) = W( y, x, 1-\alpha );$$
- $$(W_{4}) \quad d( W(x, z, \alpha ), W( y, w, \alpha ))\le (1-\alpha ) d( x, y) + \alpha d( z, w),$$

for all $x, y, z, w \in X$ and $\alpha , \beta \in [0,1]$.

It should be pointed out that if a metric space (*X*, *d*) with a mapping $W: X \times X \times [0,1] \rightarrow X$ satisfies only condition $(W_{1})$, then it coincides with the convex metric space which is introduced by Takahashi (1970) (see also Kim et al. 2007, 2008). The concept of hyperbolic spaces given here is more restrictive than the hyperbolic type defined by Goebel and Kirk (1972), since the conditions $(W_{1})- (W_{3})$ together are equivalent to (*X*, *d*) being a space of hyperbolic type in Goebel and Kirk (1972). But it is slightly more general than the hyperbolic space defined in Reich and Shafrir (1990). The class of hyperbolic spaces contains normed linear spaces and convex subsets, therefore the Hilbert ball equipped with the hyperbolic metric Goebel and Reich (1984), R-trees, Hadamard manifolds as well as CAT(0) spaces in the sense of Gromov (see Bridson and Haefliger 1999).

If $x, y \in X$ and $\lambda \in [0,1],$ then we use the notation $(1-\lambda )x \oplus \lambda y$ for $W(x, y, \lambda )$. The following holds even for the more general setting of convex metric space Takahashi (1970), for all $x, y\in X$ and $\lambda \in [0,1],$

$$\begin{aligned} d( x, (1-\lambda )x \oplus \lambda y) = \lambda d( x, y) \quad \mathrm{and}\quad d( y, (1-\lambda )x \oplus \lambda y)= ( 1-\lambda ) d( x, y). \end{aligned}$$

A hyperbolic space (*X*, *d*, *W*) is said to be uniformly convex Leuştean (2007) if for any $$ r > 0$$ and $$ \varepsilon \in (0, 2],$$ there exists $$ \delta \in (0, 1]$$ such that for all $$ a, x, y \in X,$$

$$\begin{aligned} d\bigg (\frac{1}{2} x \oplus \frac{1}{2} y, a \bigg )\le &\, {} ( 1- \delta ) r. \end{aligned}$$

provided $$ d(x, a) \le r, d(y, a) \le r $$ and $$d(x, y) \ge \varepsilon r.$$

A mapping $$ \eta :(0, \infty ) \times (0, 2] \rightarrow (0,1],$$ which providing such a $$ \delta = \eta (r, \varepsilon )$$ for given $$ r>0$$ and $$ \varepsilon \in (0, 2],$$ is called as a modulus of uniform convexity. We call the function $$\eta $$ is monotone if it decreases with *r* (for fixed $$\varepsilon $$), that is $$ \eta (r_{2} , \varepsilon ) \le \eta (r_{1}, \varepsilon ),  \forall r_{2} \ge r_{1} >0.$$

In Leuştean (2007), proved that CAT(0) spaces are uniformly convex hyperbolic spaces with modulus of uniform convexity $\eta (r, \varepsilon ) = \frac{\varepsilon ^{2}}{8}$ quadratic in $\varepsilon .$ Thus, the class of uniformly convex hyperbolic spaces are a natural generalization of both uniformly convex Banach spaces and CAT(0) spaces.

A subset $K \subset X \ne \phi$ is said to be proximal, if for each $x \in X$, there exists an element $y \in K$ such that

$$\begin{aligned} d(x, y) = {\text {dist}}( x, K) := \inf \{d( x, z): z \in K \}. \end{aligned}$$

It is well known that each weakly compact convex subset of a Banach space is proximal, as well as each closed convex subset of a uniformly convex Banach space is also proximal.

Let *CB*(*K*) be the collection of all nonempty and closed bounded subsets, and *P*(*K*) be the collection of all nonempty proximal bounded and closed subsets of *K*, respectively.

Let $H(\cdot ,\cdot )$ be the Hausdorff distance on *CB*(*K*) is defined by

$$\begin{aligned} H(A,B) = \max \bigg \{ \sup _{ x\in A} \text {dist}( x, B), \sup _{y \in B}\text {dist}( y, A) \bigg \}, ~~\forall ~A, B \in CB(X). \end{aligned}$$

Let $T: K \rightarrow 2^{K}$ be a multivalued mapping. An element $x \in K$ is said to be a fixed point of *T*, if $x \in Tx$. The set of fixed points of *T* will be denoted by *F*(*T*).

### **Definition 1**

A multivalued mapping $T: K \rightarrow CB(K)$ is said to be

(i) nonexpansive, if
  $$\begin{aligned} H(Tx, Ty) \le \, d( x, y),\quad {\text {for all}} \ \ x, y \in K, \end{aligned}$$
(ii) quasi-nonexpansive, if $F(T) \ne \phi$ and
  $$\begin{aligned} H(Tx, Tp)) \le d( x, p), \quad {\text {for all}} \ x\in K,\ {\text {and all}} \ p \in F(T). \end{aligned}$$

Now we define the multivalued version of the condition (*E*) and $(C_{\lambda })$ in the following way (see Abkar and Eslamain 2012).

### **Definition 2**

A multivalued mapping $T: K \rightarrow CB(K)$ is said to satisfy condition $(C_{\lambda })$ for some $\lambda \in (0,1)$ provided that

$$\begin{aligned} \lambda \text {dist}(x, Tx) \le d( x, y) \Rightarrow H(Tx, Ty) \le d( x, y), ~~\text {for all}~~ x, y \in K. \end{aligned}$$

### **Definition 3**

A multivalued mapping $T: K \rightarrow CB(K)$ is said to satisfy condition $(E_{\mu })$ provided that

1 $$\begin{aligned} \text {dist}(x, Ty)\le &\, {} \mu \text {dist} ( x, Tx ) + d( x, y),\quad \text {for all}~~ x, y \in K. \end{aligned}$$

We say that *T* satisfies condition (*E*) whenever *T* satisfies $(E_{\mu })$ for some $\mu \ge 1.$

We can easily prove the following proposition from the Definition 3.

### **Proposition 4**

*If*$T: K \rightarrow CB(K)$*is a multivalued mapping satisfying condition* (*E*) *with*$F(T) \ne \phi$, *thenTis a multivaluedquasi-nonexpansive mapping*.

### **Lemma 5**

Abkar and Eslamain (2012) *Let*$T: X \rightarrow CB(X)$*be a multivalued nonexpansive mapping. ThenTsatisfies condition*$(E_{1})$

Now we provide an example of generalized nonexpansive multivalued mapping satisfying condition $(C_{\lambda })$ and (*E*).

### *Example 6*

Consider $X =\{(0,0), (0,1), (1,1), (1,2) \}$ with $\ell ^{\infty }$ metric

$$\begin{aligned} d((x_{1} , y_{1}), (x_{2}, y_{2}) )= \max \{\vert x_{1}- x_{2} \vert , \vert y_{1} - y_{2} \vert \}, \end{aligned}$$

for all $$(x_{1}, y_{1})$$ and $$(x_{2}, y_{2}) $$ in *X*. Define a mapping *T* on *X* by

$$\begin{aligned} T(a, b)= \left\{ \begin{array}{ll} \{(1,1), (0,0) \}, & \quad  if \ (a, b) \ne (0,0) \\ \{(0,1) \} & \quad if \ (a, b) =(0,0). \\ \end{array} \right. \end{aligned}$$

Let $$ x=(1,2)$$ and $$ y =(0,0)$$. Then $$Tx= \{(1,1), (0,0) \}$$, $$Ty= \{(0,1)\}$$,

$$\begin{aligned} \lambda {\text {dist}} (x, Tx)= &\, \lambda {\text {dist}} ((1,2), \{(1,1), (0,0) \}) \\= &\, \lambda \le d((1,2), (0,0))=d(x,y), \end{aligned}$$

for $$ \lambda \in (0,1)$$ and

$$\begin{aligned} H(Tx, Ty)= & \,  H(\{(0,1) \}, \{(1,1), (0,0)\} ) \\= & \,  {\text {dist}}((0,1), \{(1,1), (0,0)\}) \\= & \, {} 1 \\\le & \, {} 2 \\= & \, d((0,0), (1,2))=d(x,y). \end{aligned}$$

Hence, *T* satisfy condition $(C_{\lambda }).$

On the other hand, *T* satisfy condition $$(E_{\mu })$$ for some $$\mu \ge 1.$$ In fact, take $$ x=(1,2)$$ and $$ y =(0,0)$$, then $$Ty= \{(0,1)\}$$ and $$Tx= \{(1,1), (0,0) \}.$$ Hence we have

$$\begin{aligned} {\text {dist}} (x, Ty) &=  {\text {dist}}((1,2), \{(0,1)\} ) \\ &=  1 \\ &\le  \, \mu + 2 \\ &=  \mu {\text {dist}} ((1,2),\{(1,1), (0,0) \}) +d((1,2), (0,0)) \\ &=  \mu {\text {dist}} (x, Tx) +d(x,y), \end{aligned}$$

for some $\mu \ge 1.$ This implies that *T* satisfies the condition $(C_{\lambda })$ and $(E_{\mu })$ and it is observe that *T* has unique fixed point (1, 1) in *X*. Therefore, from Proposition 4, *T* is quasi-nonexpansive.

Now, we recall the concept of $\Delta$-convergence besides collecting some of its basic properties in hyperbolic spaces.

Let $\{x_{n}\}$ be a bounded sequence in a hyperbolic space (*X*, *d*). For $x\in X$, we define a continuous functional $r_{a}(\cdot , \{x_{n}\}): X \rightarrow \mathbb {R^{+}}$ defined by

$$\begin{aligned} r_{a}( x, \{x_{n}\}) = \limsup _{n \rightarrow \infty } d( x_{n} , x), \ x \in X. \end{aligned}$$

The asymptotic radius $r(\{x_{n}\})$ of $\{x_{n}\}$ is given by

$$\begin{aligned} r(\{x_{n}\}) = \inf \{r_{a}( x, \{x_{n}\}) : x \in X\}. \end{aligned}$$

The asymptotic center $AC_{K}(\{x_{n}\})$ of a bounded sequence $\{x_{n}\}$ with respect to $K \subset X$ is the set

$$\begin{aligned} AC_{K}(\{x_{n}\}) = \{ x \in X: r_a(x, \{x_{n}\}) \le r_a(y, \{x_{n}\}), \ \ {\text {for all}} \ \ y \in K \}. \end{aligned}$$

This shows that the asymptotic center $AC_{K}(\{x_{n}\})$ of a bounded sequence is the set of minimizers of the functional $r_a(\cdot , \{x_{n}\})$ on *K*. If the asymptotic center is taken with respect to *X*,  then it is simply denoted by $AC(\{x_{n}\}).$

It is known that each uniformly convex Banach space and each CAT(0) space enjoy the property that each bounded sequence has a unique asymptotic center with respect to closed convex subsets. This property also holds in a complete uniformly convex hyperbolic space. This can be proved by Leuştean (2010) and ensures that this property is also holds in a complete uniformly convex hyperbolic space.

### **Lemma 7**

(Leuştean 2010, Proposition 3.3) *Let* (*X*, *d*, *W*) *be a complete uniformly convex hyperbolic space with monotone modulus of uniform convexity*$\eta$. *Then every bounded sequence*$\{x_{n}\}$*inXhas a unique asymptotic center with respect to any nonempty closed convex subsetKofX*.

Recall that, a sequence $\{x_{n}\}$ in *X* is said to be $\Delta$-convergent to $x \in X,$ if *x* is the unique asymptotic center of $\{u_{n}\}$ for every subsequence $\{u_{n}\}$ of $\{x_{n}\}$. In this case, we write $\Delta$-$\lim _{n \rightarrow \infty } x_{n} = x$ and call *x* the $\Delta$-limit of $\{x_{n}\}$.

### **Lemma 8**

(Khan et al. 2012) *Let* (*X*, *d*, *W*) *be a uniformly convex hyperbolic space with monotone modulus of uniform convexity*$\eta$*and*$x \in X$. *Let*$\{t_{n}\}$*be a sequence in* [*a*, *b*] *for some*$a, b \in (0,1)$. *If*$\{x_{n}\}$*and*$\{y_{n}\}$*are sequences inXsuch that*

$$\begin{aligned} \begin{aligned} \limsup _{ n \rightarrow \infty }&d( x_{n} , x) \le c , \quad \limsup _{ n \rightarrow \infty } d( y_{n} , x) \le c, \\&\lim _{ n \rightarrow \infty } d(W(x_{n} , y_{n} , t_{n} ), x ) =c, \end{aligned} \end{aligned}$$

*for some*$c \ge 0$*and*$x \in X$, *then*$\lim _{ n \rightarrow \infty } d( x_{n}, y_{n} ) =0.$

### **Lemma 9**

(Chang et al. 2015) *Let* (*X*, *d*, *W*) *be a complete uniformly convex hyperbolic space with monotone modulus of uniform convexity*$\eta$. *ThenXhas the Opial property, i.e, for any sequence*$\{x_{n}\} \subset X$*with*$\Delta$-$\lim _{n \rightarrow \infty } x_{n } = x$*and for any*$y \in X$*with*$x\ne y$, *we have*

$$\begin{aligned} \displaystyle \limsup _{n \rightarrow \infty } d( x_{n}, x) < \displaystyle \limsup _{n \rightarrow \infty } d( x_{n}, y). \end{aligned}$$

### **Lemma 10**

(Chang et al. 2014a) *Let* (*X*, *d*, *W*) *be a complete uniformly convex hyperbolic spaceXwith monotone modulus of uniform convexity*$$\eta $$*and let*$$\{x_{n}\}$$*be a bounded sequence inXwith*$$AC(\{x_{n}\})= \{p\}$$. *If*$$\{u_{n}\}$$*is a subsequence of*$$\{x_{n}\}$$*with*$$AC(\{u_{n}\}) = \{u\},$$*and the sequence*$$\{d(x_{n}, u)\}$$*is convergent, then we have*$$ p =u.$$

The following lemma is a generalization of Abkar and Eslamain (2012, Theorem 3.4) from CAT(0) space to hyperbolic space.

### **Lemma 11**

*Let* (*X*, *d*, *W*) *be a complete uniformly convex hyperbolic space with monotone modulus of uniform convexity*$\eta$, *Kbe a nonempty closed convex subset ofX*, *and*$T: K \rightarrow P(K)$*satisfies the condition* (*E*) *with convex values*. *If*$\{x_{n}\}$*is a sequence inKsuch that*$\Delta -\lim _{ n \rightarrow \infty } x_{n}= z$*and*$\lim _{ n \rightarrow \infty } \text {dist}( x_{n} , Tx_{n}) = 0,$*thenzis a fixed point ofT*.

### *Proof*

By the assumption that *Tz* is a convex and proximal subset of *K*. Hence, for each $\{x_{n}\}$ for $n \ge 1$, there exists a point $u_{z_{n}} \in Tz$ such that

$$\begin{aligned} d( x_{n} , u_{n}) = \text {dist} ( x_{n}, Tz), \quad {\text {for all}} \ \ n \ge 1. \end{aligned}$$

Taking $$ x= x_{n}$$ and $$ y = z$$ in (1), we have

2 $$\begin{aligned} \text {dist}( x_{n} , Tz)\le &\,  \mu \text {dist}( x_{n} , Tx_{n}) + d( x_{n} , z). \end{aligned}$$

Since $\{u_{n_{z}}\}$ is a bounded sequence in *Tz*, by Lemma 7, there exists a subsequence $\{u_{z_{n_{k}}}\} \subset \{u_{z_{n}}\}$ such that $\Delta -\lim _{ k \rightarrow \infty } u_{z_{n_{k}}} = u_{z} \in Tz.$ Since *T* satisfies the condition (*E*), we have

$$\begin{aligned} d( x_{n_{k}}, u_{z})\le & \, {} d( x_{n_{k}}, u_{z_{n}}) + d( u_{z_{n}}, u_{z}) \\\le & \,{} \mu \text {dist} (x_{n_{k}}, Tx_{n_{k}}) + d( x_{n_{k}}, z) + d( u_{n} , u_{z}). \end{aligned}$$

Taking the superior limit on the both sides of the above inequality, we have

$$\begin{aligned} \limsup \limits _{n\rightarrow \infty } d( x_{n_{k}}, u_{z})\le & {} \limsup \limits _{n\rightarrow \infty } \bigg \{ \mu \text {dist} (x_{n_{k}}, Tx_{n_{k}}) + d( x_{n_{k}}, z) + d( u_{n} , u_{z}) \bigg \} \\< & {} \limsup \limits _{n\rightarrow \infty } d( x_{n_{k}}, u_{z}). \end{aligned}$$

Hence by Lemma 9 $u_{z} = z \in Tz$ and the proof is completed. $\square$

## Main results

In this section, we established $\Delta$-convergence and strong convergence theorems for the iterative sequence introduced by Chang et al. (2014a) (see the single valued version, Kim et al. 2015; Kim and Dashputre 2015; Agarwal et al. 2007).

### **Theorem 12**

*Let* (*X*, *d*, *W*) *be a complete uniformly convex hyperbolic space with monotone modulus convexity*$\eta$*andKbe a nonempty closed convex subset ofX*. *Let*$T: K \rightarrow P(K)$*be a multivalued mapping satisfying the condition* (*E*) *with convex values. Suppose that*$F(T) \ne \phi$*and*$Tp = \{p\}$*for each*$p \in F(T).$*For arbitrarily chosen*$x_{0} \in K$, *sequence*$\{x_{n}\}$*is defined by*

3 $$\begin{aligned}&\left\{ \begin{array}{ll} x_{n+1} = W(u_{n} , v_{n} , \alpha _{n}), \\ y_{n} = W( x_{n} , u_{n} , \beta _{n}), \\ \end{array} \right. \end{aligned}$$

*where*$v_{n} \in Ty_{n}, u_{n} \in Tx_{n}, \{\alpha _{n}\}$*and*$\{\beta _{n} \}$*are real sequences satisfying the following condition:*

($$C_{1}$$)
*There exists constants*$a, b \in (0,1)$*with*$0< b(1-a)\le \frac{1}{2}$*such that*$\{\alpha _{n}\} \subset [a, b]$*and*$\{\beta _{n}\} \subset [a, b].$

*Then the sequence*$\{x_{n}\}$*defined by* (3) *is*$\Delta$-*convergent to a point inF*(*T*).

### *Proof*

The proof of Theorem 12 is divided into three steps as follows:

**Step-I**. First, we prove that $\lim _{n \rightarrow \infty } d( x_{n} , p)$ exists for $p\in F(T)$.

In fact, from Proposition 4, *T* is a multivalued quasi-nonexpansive mapping. Hence for each $p \in F(T)$, by (3), we have

4 $$\begin{aligned} d( y_{n}, p)= &\,  d(W(x_{n} , u_{n} , \beta _{n}), p) \nonumber \\\le & \, ( 1- \beta _{n}) d( x_{n} , p) + \beta _{n} d( u_{n}, p) \nonumber \\\le & \, ( 1-\beta _{n}) d(x_{n}, p) + \beta _{n} {\text {dist}}(u_{n} , Tp) \\\le & \, ( 1- \beta _{n}) d(x_{n}, p) + H(Tx_{n},Tp) \nonumber \\\le & \, ( 1-\beta _{n}) d(x_{n} , p) + \beta _{n} d( x_{n} , p) \nonumber \\\le & \, d( x_{n} , p). \nonumber \end{aligned}$$

Again from (3) and (4), we have

5 $$\begin{aligned} d( x_{n+1} , p)= & \, d( W( u_{n} , v_{n}, \alpha _{n}, p)) \nonumber \\\le & \, (1-\alpha _{n}) d( u_{n} , p) + \alpha _{n} d( v_{n} , p) \nonumber \\\le & \, (1-\alpha _{n}) {\text {dist}}( u_{n} , Tp) + \alpha _{n} {\text {dist}}(v_{n} , Tp) \nonumber \\\le & \, (1-\alpha _{n}) H(Tx_{n} , Tp) + \alpha _{n} H(Ty_{n}, Tp) \\\le & \, (1-\alpha _{n}) d( x_{n} , p) + \alpha _{n} d( y_{n} , p) \nonumber \\\le & \, (1-\alpha _{n}) d( x_{n} , p) + \alpha _{n} d( x_{n} , p) \nonumber \\\le & \, d( x_{n}, p).\nonumber \end{aligned}$$

This shows that sequence $\{d( x_{n} , p)\}$ is decreasing and bounded below. Hence the $\lim _{ n \rightarrow \infty } d( x_{n} , p)$ exists for each $p \in F(T).$

**Step-II**. Next, we prove that

$$\begin{aligned} \lim _{ n \rightarrow \infty } {\text {dist}}( x_{n} , Tx_{n} ) = 0. \end{aligned}$$

From the Step-I, we know that for each $$ p \in F(T)$$, $$\lim _{n \rightarrow \infty } d( x_{n}, p) $$ exists, Let $$ \lim _{n \rightarrow \infty } d( x_{n}, p) = c.$$ If $$ c=0$$, then we have

$$\begin{aligned} {\text {dist}}( x_{n} , Tx_{n})\le & \, d( x_{n} , p) + {\text {dist}}( Tx_{n} , Tp) \\\le & \, d(x_{n} , p) + H(Tx_{n}, Tp) \\\le & \, 2 d( x_{n} , p) \\\rightarrow & \, 0, ~~{\text {as}}~~ n \rightarrow \infty . \end{aligned}$$

Hence, the conclusion holds for $$ c=0.$$ If $$ c >0$$. Letting $$ n \rightarrow \infty $$ and taking superior limit to the both sides of inequality (4), we have

6 $$\begin{aligned} \limsup _{n \rightarrow \infty } d( y_{n} , p)\le & \, c. \end{aligned}$$

In addition, we have

$$\begin{aligned} d( u_{n}, p)= & \, {\text {dist}}(u_{n}, Tp) \le H(Tx_{n}, Tp) \le d( x_{n}, p). \end{aligned}$$

Letting $n \rightarrow \infty$ and taking superior limit to the both sides of above inequality, we have

7 $$\begin{aligned} \limsup _{n \rightarrow \infty } d( u_{n}, p)\le & \, c. \end{aligned}$$

In similar lines, we have

8 $$\begin{aligned} \limsup _{n \rightarrow \infty } d( v_{n}, p)\le & \, c, \end{aligned}$$

Since $\lim _{ n \rightarrow \infty } d( x_{n+1} , p) =c,$ it follows from (7) and (8) and using Lemma 8, we have

9 $$\begin{aligned} \displaystyle \lim _{n \rightarrow \infty } d( u_{n} , v_{n})= & \, 0. \end{aligned}$$

Further, by (3), we have

$$\begin{aligned} d( x_{n+1}, p)= & \, d(W( u_{n} , v_{n} , \alpha _{n}), p) \\\le & \, \alpha _{n} d( u_{n} , p) + (1- \alpha _{n}) d(v_{n} ,p) \\\le & \, \alpha _{n} {\text {dist}}(u_{n} , Tp) + ( 1- \alpha _{n}) {\text {dist}}( v_{n} , Tp) \\\le & \, \alpha _{n} H(Tx_{n},Tp) + ( 1-\alpha _{n}) H(Ty_{n}, Tp) \\\le & \, ( 1- \alpha _{n}) d( y_{n} , p) + \alpha _{n} d(x_{n} , p) \end{aligned}$$

or

$$\begin{aligned} ( 1- \alpha _{n}) d( x_{n+1}, p)\le & \, (1-\alpha _{n}) d( y_{n} , p) + \alpha _{n}\big \{d( x_{n}, p) - d(x_{n+1}, p)\big \} \\\le & \, d( y_{n} , p) + \frac{b}{1-b}\bigg \{d( x_{n} , p) - d( x_{n+1}, p)\bigg \}. \end{aligned}$$

Letting $n \rightarrow \infty$ and taking inferior limit to the both sides of the above inequality, we have

10 $$\begin{aligned} c\le & \, \liminf _{ n \rightarrow \infty } d(y_{n} , p). \end{aligned}$$

Hence, from (6) and (10), we have

$$\begin{aligned} \displaystyle \lim _{n \rightarrow \infty } d( y_{n}, p)= & c. \end{aligned}$$

By using (6) and apply Lemma 8, then we have

11 $$\begin{aligned} \displaystyle \lim _{ n \rightarrow \infty } d( u_{n} , x_{n})= &  0, \end{aligned}$$

it implies that

$$\begin{aligned} \displaystyle \lim _{ n \rightarrow \infty } {\text {dist}}(x_{n}, Tx_{n}) =0. \end{aligned}$$

**Step-III**. Finally, in order to show that the sequence $\{x_{n}\}$ is $\Delta$-convergent to a point in *F*(*T*), we prove that

$$\begin{aligned} W_{\Delta } (\{x_{n}\}) := \bigcup _{\{u_{n}\} \subset \{x_{n}\}} AC(\{u_{n}\}) \subset F(T) \end{aligned}$$

and $W_{\Delta } (\{x_{n}\})$ consists of exactly one point.

Let $$ u \in W_{\Delta } (\{x_{n}\})$$. Then there exists a subsequence $$\{u_{n}\}$$ of $$ \{x_{n}\}$$ such that $$AC( \{u_{n}\})= \{u\}$$. Then by Lemma 7, there exists a subsequence $$\{v_{n}\}$$ of $$\{u_{n}\}$$ such that $$\Delta -\lim _{n \rightarrow \infty } v_{n}= v \in K. $$ Since $$\lim _{n \rightarrow \infty } {\text {dist}} (v_{n} , Tv_{n}) =0$$, from Lemma 11, we have $$ v \in F(T).$$ Since $$\{d(u_{n} , v) \}$$ converges, from Lemma 10, we have $$ u= v.$$ This shows that $$ W_{\Delta } (x_{n}) \subset F(T).$$

Next, we claim that $$ W_{\Delta } (\{x_{n}\})$$ is a singleton set. Let $$\{u_{n}\}$$ be a subsequence of $$\{x_{n}\}$$ with $$AC(\{u_{n}\}) = \{u\}$$ and $$AC(\{x_{n}\})= \{x\}$$. We have already seen that $$ u = v$$ and $$ v \in F(T)$$. Finally, since $$\{d(x_{n} , p)\}$$ is convergent, from Lemma 10, we have $$ x = v \in F(T).$$ This shows that $$W_{\Delta }(\{x_{n}\}) = \{x\}$$ and completes the proof. $$\square $$

### **Theorem 13**

Let (*X*, *d*, *W*) be a complete uniformly convex hyperbolic space with monotone modulus convexity $\eta$ and *K* be a nonempty compact convex subset of *X*. Let $T: K \rightarrow CB(X)$ be a multivalued mapping satisfying the condition (*E*) with nonempty convex values. Let $F(T) \ne \phi$ and $Tp = \{p\}$ for each $p \in F(T).$ Then the iterative process $\{x_{n}\}$ defined by (3) converges strongly to a point in *F*(*T*).

### *Proof*

By the assumption, we know that each $x \in K$ and *Tx* is a bounded closed and convex subset of *K*. Since *K* is compact, *Tx* is a nonempty compact convex subset and a bounded proximal subset in *K*, i.e., $T: K \rightarrow P(K)$. Therefore all the conditions of Theorem 12 are satisfied. Hence, it follows from Theorem 12 that

$$\begin{aligned} \lim _{n \rightarrow \infty } d (x_{n} , p)\ \ \ {\text {exists, }} \ \ {\text {and}} \ \ \lim _{n \rightarrow \infty } \text {dist} ( x_{n}, Tx_{n}) =0. \end{aligned}$$

Furthermore, since *K* is compact, there exists a subsequence $\{x_{n_{k}}\}$ of $\{x_{n}\}$ such that $\lim _{n \rightarrow \infty } x_{n_{k}} = \omega \in K.$ By condition (*E*), we have, for some $\mu \ge 1$

$$\begin{aligned} {\text {dist}}( \omega , T\omega )\le & \, d ( \omega , x_{n_{k}}) + {\text {dist}} ( x_{n_{k}}, T\omega ) \\\le & \, \mu {\text {dist}}( x_{n_{k}}, Tx_{n_{k}})+ 2 d( \omega , x_{n_{k}}) \\\rightarrow & \, 0, ~~{\text {as}}~~k \rightarrow \infty . \end{aligned}$$

This implies that $$ \omega \in F(T) $$. Since $$\{x_{n_{k}}\}$$ converges strongly to $$ \omega $$ and the $$ \lim _{ n\rightarrow \infty } d( x_{n} , \omega )$$ exists from Theorem 12, the sequence $$\{x_{n}\}$$ converges strongly to $$\omega .$$$$\square $$

### **Theorem 14**

*Let* (*X*, *d*, *W*) *be a complete uniformly convex hyperbolic space with monotone modulus convexity*$\eta$*andKbe a nonempty compact convex subset ofX*. *Let*$T: K \rightarrow CB(X)$*be a multivalued mapping satisfying the condition* (*E*) *with nonempty convex values*. *Let*$F(T) \ne \phi$*and*$Tp = \{p\}$*for each*$p \in F(T).$*Then the iterative process*$\{x_{n}\}$*defined by* (3) *converges strongly to a point inF*(*T*) *if and only if*

$$\begin{aligned} \liminf _{n \rightarrow \infty } {\text {dist}}( x_{n}, F(T))=0. \end{aligned}$$

### *Proof*

Necessity is obvious. To prove the sufficiency, suppose that

$$\begin{aligned} \liminf _{n \rightarrow \infty } {\text {dist}}( x_{n}, F(T))=0. \end{aligned}$$

From (4), we have

$$\begin{aligned} d( x_{n+1} , p)\le & \, d( x_{n}, p) \end{aligned}$$

for all $p \in F(T)$. This implies that

$$\begin{aligned} {\text {dist}} ( x_{n+1}, F(T))\le & \, {\text {dist}} ( x_{n}, F(T)). \end{aligned}$$

Hence the limit $\lim _{n \rightarrow \infty } {\text {dist}} ( x_{n+1}, F(T))$ exists and $\lim _{n \rightarrow \infty } {\text {dist}} ( x_{n+1}, F(T))=0.$ Therefore, we can choose a subsequence $\{x_{n_{k}}\}$ of $\{x_{n}\}$ and a sequence $\{p_{k}\}$ in *F*(*T*) such that for all $k \in \mathbb {N},$

$$\begin{aligned} d( x_{n_{k}}, p_{k})\le & \, \frac{1}{2^{k}}. \end{aligned}$$

From (4), we have

$$\begin{aligned} d( x_{n_{k+1}}, p_{k})\le & \, d(x_{n_{k}}, p_{k}) < \frac{1}{2^{k}}. \end{aligned}$$

Hence

$$\begin{aligned} d( p_{k+1}, p_{k})\le & \, d( x_{n_{k+1}}, p_{k+1}) + d( x_{n_{k+1}}, p_{k}) \\<&\frac{1}{2^{k+1}}+ \frac{1}{2^{k}} \\< & {} \frac{1}{2^{k-1}}. \end{aligned}$$

Consequently, $\{p_{k}\}$ is a Cauchy sequence in *K* and it is convergent to some $q \in K$. Since

$$\begin{aligned} {\text {dist}}( p_{k}, Tq)\le & \, H(Tp_{k}, Tq) \le d( q, p_{k}) \end{aligned}$$

and $p_{k} \rightarrow q$ as $k \rightarrow \infty$, it follows that ${\text {dist}}(q, Tq)=0$ , and hence $q \in F(T)$ and $\{x_{n_{k}}\}$ converges strongly to *q*. Since $\lim _{ n \rightarrow \infty } d( x_{n} , q)$ exists, it follows that $\{x_{n}\}$ converges strongly to *q*. $\square$

### **Theorem 15**

*Let* (*X*, *d*, *W*) *be a complete uniformly convex hyperbolic space with monotone modulus convexity*$$\eta $$*andKbe a nonempty compact convex subset ofX*. *Let*$$T: K \rightarrow CB(X)$$*be a multivalued mapping satisfying the condition* (*E*) *with nonempty convex values*. *Let*$$ F(T) \ne \phi $$*and*$$Tp = \{p\}$$*for each*$$ p \in F(T)$$. *Let*$$\{x_{n}\}$$*be the iterative process defined by* (3). *Suppose that there exists an increasing function*$$ f: [0,\infty ) \rightarrow [0, \infty )$$*with*$$f(0) =0$$, *where*$$ f(r) > 0$$*for all*$$ r >0$$*such that*

$$\begin{aligned} {\text {dist}}( x_{n} , Tx_{n})\, \ge\, f ({\text {dist}} ( x_{n} , F(T) ). \end{aligned}$$

*Then the sequence*$\{x_{n}\}$*converges strongly to a point ofF*(*T*).

### *Proof*

By Theorem 12, we have ${\text {dist}}( x_{n} , Tx_{n}) =0$. Hence from the assumption, we have

$$\begin{aligned} \displaystyle \lim _{n \rightarrow \infty } f ({\text {dist}} ( x_{n} , F(T) )\le & {} \displaystyle \lim _{ n\rightarrow \infty } {\text {dist}}( x_{n} , Tx_{n}) =0. \end{aligned}$$

Therefore, we have

$$\begin{aligned} \displaystyle \lim _{n \rightarrow \infty } f ({\text {dist}} ( x_{n} , F(T))=0. \end{aligned}$$

Since $f: [0,\infty ) \rightarrow [0, \infty )$ is an increasing function with $f(0) =0$, we we obtain $\lim _{n \rightarrow \infty } {\text {dist}}( x_{n}, F(T))=0.$ The rest of the proof follows in the lines of Theorem 14.

We need the following lemma for the proof of the $$\Delta $$-convergence theorem. We can easily prove this lemma from the definition of $$P_T x.$$$$\square $$

### **Lemma 16**

*Let* (*X*, *d*, *W*) *be a complete uniformly convex hyperbolic space*, *Kbe a nonempty closed convex subset ofX*. *Let*$T: K \rightarrow P(K)$*be a multivalued mapping with*$F(T) \ne \phi$*and let*$P_{T} : K \rightarrow 2^{K}$*be a multivalued mapping defined by*

12 $$\begin{aligned} P_{T}x= & \, \{ y \in Tx: d( x, y) = {\text {dist}}( x, Tx)\}, \quad x \in K. \end{aligned}$$

*Then the following conclusions are hold:*

1. $$F(T) = F(P_{T});$$
2. $$ P_{T}p = \{p\}$$, *for each*$$ p \in F(T);$$
3. *For each*$x \in K$, $P_{T}x$*is a closed subset ofTxand so it is compact*;
4. $$ d( x, Tx) = d( x, P_{T}x),$$*for each*$$ x \in K;$$
5. $P_{T}$*is a multivalued mapping fromKtoP*(*K*).

### **Theorem 17**

*Let* (*X*, *d*, *W*) *be a complete uniformly convex hyperbolic space with monotone modulus of uniform convexity*$\eta$*andKbe a nonempty closed convex subset ofX*. *Let*$T: K \rightarrow CB(K)$*multivalued mapping with convex values and*$F(T)\ne \phi$. *Let*$P_{T}$*be a multivalued mapping satisfying the condition* (*E*). *Let for arbitrarily choose*$x_{0} \in K$, $\{x_{n}\}$*be a sequence defined by*

13 $$\begin{aligned}&\left\{ \begin{array}{ll} &x_{n+1} = W(u_{n} , v_{n} , \alpha _{n}), \\ &y_{n} = W( x_{n} , u_{n} , \beta _{n}), \\ \end{array} \right. \end{aligned}$$

*where*$u_{n} \in P_{T}x_{n}, v_{n} \in P_{T}y_{n} = P_{T} ((W( x_{n} , u_{n}, \beta _{n})),$*let*$\{\alpha _{n}\}$*and*$\{\beta _{n} \}$*be real sequences satisfying the condition*$(C_{1})$. *Then the sequence*$\{x_{n}\}$*defined by* (13) *is*$\Delta$-*convergent to a point inF*(*T*).

### *Proof*

In the view of Lemma 16, we know that the mapping $P_{T}$ defined by (12) has the following property, that is, for each $p \in F(T),$ we have

$$\begin{aligned} P_{T}p = \{y \in Tp: d( p, y) = dist( p , Tp) =0 \}= \{p\}. \end{aligned}$$

Replacing mapping *T* by $P_{T}$ in Theorem 12, then all the conditions in Theorem 12 are satisfied. Therefore the conclusion of Theorem 17 can be obtained in the same line of Theorem 12. $\square$

### **Theorem 18**

*Let* (*X*, *d*, *W*) *be a complete uniformly convex hyperbolic space andKbe a nonempty closed convex subset ofXwith monotone modulus of uniform convexity*$\eta$. *Let*$T: K \rightarrow CB(K)$*be a multivalued mapping with convex values and*$F(T)\ne \phi$. *Let*$P_{T}$*be a multivalued mapping satisfying the condition* (*E*). *For arbitrarily*$x_{0} \in K,$*let*$\{x_{n}\}$*be a sequence inKdefined by* (13). *Then the sequence*$\{x_{n}\}$*converges strongly to*$p \in F(T)$*if and only if*

$$\begin{aligned} \liminf _{ n \rightarrow \infty } dist( x_{n} ,F(T))=0. \end{aligned}$$

### *Proof*

If $\{x_{n}\}$ converges to $p \in F(T)$, then $\lim _{ n \rightarrow \infty } d( x_{n} , p) =0.$ Since $0\le dist( x_{n} , F(T)) \le d( x_{n} , p),$ we have

$$\begin{aligned} \liminf _{ n \rightarrow \infty } dist( x_{n} , F(T)) =0. \end{aligned}$$

Conversely, if we assume that $\liminf _{ n \rightarrow \infty } dist( x_{n} , F(T)) =0.$ Let $p \in F(T)$. Then we have

$$\begin{aligned} P_{T}p = \{y \in Tp: d( p , y) = d( p , Tp) =0 \}= \{p\}. \end{aligned}$$

Moreover, by the same method as given in the proof of Theorem 12, we can see that

$$\begin{aligned} dist(x_{n+1} , F(T)) \le d( x_{n} , F(T)), \end{aligned}$$

it implies that $\lim _{ n \rightarrow \infty } dist( x_{n} , F(T))$ exists. Hence from hypothesis, we have $\liminf _{n \rightarrow \infty } dist( x_{n} , F(T))=0.$ Therefore, we can choose a subsequence $\{x_{n_{k}}\}$ of $\{x_{n}\}$ and a sequence $\{p_{k}\}$ in *F*(*T*) such that for all $k \in \mathbb {N}$

$$\begin{aligned} d( x_{n_{k}}, p_{k}) < \frac{1}{2^k}. \end{aligned}$$

As in proof of Theorem 14, we show that $\{p_{k}\}$ is a Cauchy sequence in K and hence converges to some $q \in K$. By the virtue of the definition of mapping $P_{T}$, we have $P_{T} q \subset Tq$. Hence from (12), we have

$$\begin{aligned} {\text {dist}}(p_{k}, Tq)\le & \, {\text {dist}}( p_{k} , P_{T}q) \\\le & \, H(P_{T}p_{k}, P_{T}q) \\\le & \, d(q, p_{k}), \end{aligned}$$

$p_{k} \rightarrow q$ as $k \rightarrow \infty ,$ it follows that ${\text {dist}} ( q, Tq) =0.$ Hence $q \in F(T)$ and $\{x_{n_{k}}\}$ converges strongly to *q*. Since $\lim _{ n \rightarrow \infty } d( x_{n} , p)$ exists, we conclude that $\{x_{n}\}$ converges strongly to *q* . This completes the proof.$\square$

### **Theorem 19**

*Let* (*X*, *d*, *W*) *be a complete uniformly convex hyperbolic space andKbe a nonempty closed convex subset ofXwith monotone modulus of uniform convexity*$$ \eta $$. *Let*$$T: K \rightarrow CB(K)$$*multivalued mapping with convex values and*$$ F(T)\ne \phi $$. *Let*$$P_{T}$$*be a multivalued mapping satisfying the condition* (*E*). *Let*$$\{x_{n}\}$$*be a sequence inKdefined by* (13). *Assuming that there exists an increasing function*$$f:[0, \infty ) \rightarrow [0, \infty )$$*with*$$f(0)=0$$*and*$$f(r) >0$$*for all*$$ r >0$$*such that*

$$\begin{aligned} {\text {dist}}(x_{n} , Tx_{n})\ge & \, f({\text {dist}}(x_{n} ,F(T))). \end{aligned}$$

*Then the sequence*$\{x_{n}\}$*converges strongly to a fixed point inF*(*T*).

### *Proof*

By Theorem 12, we have ${\text {dist}}( x_{n} , Tx_{n}) =0$. Hence from the assumption that

$$\begin{aligned} \displaystyle \lim _{n \rightarrow \infty } f ({\text {dist}} ( x_{n} , F(T)) )\le & {} \displaystyle \lim _{ n\rightarrow \infty } {\text {dist}}( x_{n} , Tx_{n}) =0. \end{aligned}$$

Therefore, we have

$$\begin{aligned} \displaystyle \lim _{n \rightarrow \infty } f ({\text {dist}} ( x_{n} , F(T)) )=0. \end{aligned}$$

Since $f: [0,\infty ) \rightarrow [0, \infty )$ is an increasing function with $f(0) =0$, we we obtain $\lim _{n \rightarrow \infty } {\text {dist}}( x_{n}, F(T))=0.$ The rest of the proof follows in the lines of Theorem 18. $\square$

## Numerical example

Let $(X, d)= \mathbb {R}$ with metric $d( x, y) = \vert x - y \vert$ and $K = [0,5]$. Denoted by

$$\begin{aligned} W( x, y, \alpha ) = \alpha x + ( 1- \alpha ) y, \end{aligned}$$

for all $x, y \in X$ and $\alpha \in [0,1],$ then (*X*, *d*, *W*) is a complete uniformly convex hyperbolic space with a monotone modulus of uniform convexity and K is a nonempty closed and convex subset of X. Let $T: K \rightarrow P(K)$ be a multivalued mapping defined by

$$\begin{aligned} T(x)= \left\{ \begin{array}{ll} [0, \frac{x}{5} ], \quad x \ne 5, \\ \{1\}, \quad x = 5. \\ \end{array} \right. \end{aligned}$$

Then it is easy to prove that (also see Abkar and Eslamain 2012), $T: K \rightarrow P(K)$ is a generalized nonexpansive multivalued mapping satisfying the condition, $(C_{\lambda })$ and (*E*) with convex value $0\in K$ which is a unique fixed point in *K* and $T(0) =\{0\}.$ Therefore *T* is a generalized nonexpansive multivalued mapping satisfying the condition (*E*) and satisfies all conditions in Theorem 12. Let $\{\alpha _{n}\}$ and $\{\beta _{n}\}$ be two constant sequences such that $\{\alpha _{n}\} =\{\beta _{n}\} =\frac{1}{2}$ for all $n \ge 0.$ For any given $x_{0} \in [0,5]$ (for the sake of simplicity, we can assume that $x_{0} =1$). Using (3), we have, take $x_{0} =1$, we have $Tx_{0} = [ 0, \frac{1}{5}]$, taking $u_{0} = \frac{1}{5}\in Tx_{0},$ then

$$\begin{aligned} y_{0}= & \, W\bigg (x_{0}, u_{0}, \frac{1}{2}\bigg ) = \frac{1}{2}\bigg (1 + \frac{1}{5} \bigg ). \end{aligned}$$

Now we compute $Ty_{0} = \bigg [0, \frac{\frac{1}{2}(1+\frac{1}{5} )}{5} \bigg ]$, taking $v_{0} =\frac{1}{2 \times 5^{2}} \in Ty_{0},$ then

$$\begin{aligned} x_{1}= &\, W\bigg (u_{0}, v_{0}, \frac{1}{2} \bigg )= \frac{1}{2 \times 5}\bigg [1+ \frac{1}{2 \times 5} \bigg ]. \end{aligned}$$

From the definition of *T*, we have $Tx_{1} = \bigg [0, \frac{\frac{1}{2 \times 5}\big (1+ \frac{1}{2 \times 5} \big )}{5} \bigg ]$, taking $u_{1} = \frac{1}{2^{2} \times 5^{3}} \in Tx_{1}$, then

$$\begin{aligned} y_{1}= & \, W \bigg (u_{1}, x_{1}, \frac{1}{2} \bigg ) \\= & \, \frac{1}{2^{2} \times 5}\bigg [1+ \frac{1}{2 \times 5}+ \frac{1}{2 \times 5^{2}} \bigg ]. \end{aligned}$$

Hence, $Ty_{1} = \bigg [0, \frac{ \frac{1}{2^{2} \times 5}\big (1+ \frac{1}{2 \times 5}+ \frac{1}{2 \times 5^{2}} \big )}{5} \bigg ]$, taking $v_{1} = \frac{1}{2^{3} \times 5^{4}}$. Hence,

$$\begin{aligned} x_{2}= & \, W\bigg (u_{1}, v_{1}, \frac{1}{2} \bigg ) \\= & \, \frac{1}{2^{3} \times 5^{3}} \bigg (1+ \frac{1}{2 \times 5} \bigg ). \end{aligned}$$

Inductively, we have

$$\begin{aligned} x_{n+1}= & {} \frac{1}{2^{2n+1} \times 5^{2n+1}} \bigg (1+ \frac{1}{2 \times 5} \bigg ). \end{aligned}$$

Hence we have $\lim _{n \rightarrow \infty } x_{n} =0 \in F(T).$

## Conclusion remarks

Our Theorems 12, 13, 14, 15, 17, 18 and 19 are improvements and extensions of the corresponding results in the following senses:

1. Our results are setting in hyperbolic spaces instead of uniformly convex Banach spaces in Chang et al. (2014b, Theorems 3.1,  3.2 and Theorems 4.1, 4.2 and 4.3) and Khan et al. (2012, Theorems 1 and 2).
2. We used the generalized multivalued mappings in hyperbolic spaces instead of the SCC, SKC, KSC, KSC SCS and *C*-type multivalued mappings in Chang et al. (2015, Theorems 2.1 and  2.4).
3. We used the generalized nonexpansive multivalued mappings in hyperbolic spaces instead of nonexpansive multivalued mappings in Chang et al. (2014a, Theorems 2.1 and 2.2) and Khan et al. (2014, Theorems 2.4 and 2.5)
4. Our results are setting in hyperbolic spaces instead of CAT(0) spaces in Abkar et al. (2012, Theorems 3.6, 3.7 and 3.12) and Pathak et al. (2015, Theorems 3.2, 3.3 and 3.4).
